# The Role of Mitochondrial Quality Control in Cognitive Dysfunction in Diabetes

**DOI:** 10.1007/s11064-022-03631-y

**Published:** 2022-06-04

**Authors:** Jian-Sheng Luo, Jia-Qi Ning, Zhuo-Ya Chen, Wen-Jing Li, Rui-Ling Zhou, Ru-Yu Yan, Meng-Jie Chen, Ling-Ling Ding

**Affiliations:** Beijing Hospital of Traditional Chinese Medicine, Capital Medical University, Beijing, 100010 China

**Keywords:** Mitochondrial quality control, Mitochondria, Cognitive dysfunction, Diabetes

## Abstract

Type 2 diabetes (T2DM) is a well known risk factor for Alzheimer’s disease. Mitochondria are the center of intracellular energy metabolism and the main source of reactive oxygen species. Mitochondrial dysfunction has been identified as a key factor in diabetes-associated brain alterations contributing to neurodegenerative events. Defective insulin signaling may act in concert with neurodegenerative mechanisms leading to abnormalities in mitochondrial structure and function. Mitochondrial dysfunction triggers neuronal energy exhaustion and oxidative stress, leading to brain neuronal damage and cognitive impairment. The normality of mitochondrial function is basically maintained by mitochondrial quality control mechanisms. In T2DM, defects in the mitochondrial quality control pathway in the brain have been found to lead to mitochondrial dysfunction and cognitive impairment. Here, we discuss the association of mitochondrial dysfunction with T2DM and cognitive impairment. We also review the molecular mechanisms of mitochondrial quality control and impacts of mitochondrial quality control on the progression of cognitive impairment in T2DM.

## Introduction

It is estimated that there are 537 million people living with diabetes worldwide and this number is expected to over 783 million in 2045, due to the aging population and increased longevity [1]. Type 2 diabetes (T2DM) is a major subtype of diabetes, accounting for more than 90% of all diabetes cases [2]. T2DM is characterized by relative insulin deficiency caused by pancreatic β-cell dysfunction and insulin resistance in target organs [3, 4]. Clinical studies have found that T2DM causes atrophy in frontal and temporal regions of the brain, especially the hippocampus, which is associated with poorer visuospatial construction, executive function, and memory function [5–7]. It has been found that T2DM increases the risk of multiple forms of cognitive impairment, including Alzheimer’s disease (AD) [8, 9]. A meta-analysis suggested that patients with diabetes had a 56% increased risk of AD [10].

The cognitive changes associated with T2DM appear to begin in the prediabetic phase of insulin resistance [11]. The brain is an organ susceptible to insulin. It is rich in insulin receptors in areas closely related to cognitive memory, such as the hippocampus [12]. Studies have shown that decreased brain sensitivity to insulin can lead to energy failure and ad like pathological changes, including amyloid beta peptide (Aβ) deposition and aberrant tau phosphorylation, leading to impaired nerve growth, synaptic plasticity, and cognitive function [13–15]. This suggests an association between AD and T2DM. In fact, AD is considered a brain-specific form of diabetes. AD patients have shown reduced brain insulin receptor sensitivity, hyperphosphorylation of insulin receptor and downstream second messenger such as insulin receptor substrate-1 (IRS-1) [16–18]. Cognitive function improved after treatment with insulin sensitizer or intranasal insulin in patients with AD [19–21].

The cognitive function of the brain depends on synaptic communication between neurons. This leads to high levels of energy demand, so synapses are rich in mitochondria [22, 23]. Mitochondria are also a major source and target of intracellular reactive oxygen species (ROS). Multiple studies have shown that mitochondrial dysfunction may be a key player in diabetes-associated brain alterations contributing to neurodegenerative events [24–26]. In the insulin resistant brain, the structural and functional damage of brain mitochondria was observed, including reduced mitochondrial electron transport chain (mETC) activity, decreased mitochondrial respiration and massive production of ROS [27–30]. This not only leads to energy exhaustion, but also oxidative stress. Uncontrolled oxidative stress can promote the accumulation of Aβ in synaptic mitochondria, induce neuronal apoptosis and lead to cognitive impairment [30–33].

Numerous quality control mechanisms have evolved within mitochondria to maintain proper function basally and in response to stress, including proteostasis, biogenesis, dynamics, and mitophagy [34]. Mitochondrial protein import is controlled and occurs in their unfolded form through various translocases, which is supported by mitochondrial membrane potential [35, 36]. Molecular chaperones and intramitochondrial proteases control the integrity and proper assembly of the imported proteins [37, 38]. In addition, parts of or even entire mitochondria can be removed via several mechanisms as discussed later [39, 40]. In this review, we focus on mitochondrial dysfunction, and summarize current knowledge of the role of mitochondrial quality control mechanisms in cognitive dysfunction in diabetes.

### Mitochondrial Dysfunction as a Link Between T2DM and AD

Studies have shown that a high-fat diet results in an increased flux of free fatty acids into circulation, which are absorbed by the liver or skeletal muscle for beta-oxidation in the mitochondria or stored as triglycerides [41, 42]. When fatty acid flux exceeds the processing capacity of these pathways, fatty acid metabolic intermediates (particularly ceramide) will accumulate [43, 44]. Although the role of ceramide in the formation of insulin resistance is contentious, some evidence suggests that ceramide may contribute to brain insulin resistance [45–51]. Ceramide can enter the brain through the blood–brain barrier, and can also be produced in the brain through de novo synthesis or sphingomyelin hydrolysis [50–53]. In canonical insulin signaling, insulin binds to insulin receptor, inducing IR autophosphorylation, recruitment of insulin receptor substrate (IRS) adaptor proteins, and then activates phosphatidylinositol 3-kinase (PI3K)/AKT pathway, thereby exerting a variety of anabolic activities [54]. Ceramide can activate c-Jun N-terminal kinase (JNK) and IκB kinase to inactivate IRS-1 phosphorylation, and also inhibit the PI3-K/Akt pathway through protein phosphatase 2A dephosphorylation, leading to insulin signaling disruption and neuronal apoptosis [55, 56]. Interestingly, exposure to ceramide also leads to neuronal mitochondrial dysfunction. However, the role of mitochondrial dysfunction in the formation of brain insulin resistance is largely unknown, and few studies suggest that mitochondrial dysfunction contributes to brain insulin resistance [27, 57]. A study has shown that high glucose induces neuronal mitochondrial dysfunction, and subsequent mitochondrial dysfunction leads to impairment of the AMPK/Akt pathway, which is part of the insulin pathway and may lead to insulin resistance [57].

Insulin signaling has important implications for brain mitochondrial function [58, 59]. PI3-K/Akt can activate glucose transporter 3 to promote glucose uptake in neurons, and induce hexokinase II to bind to the mitochondrial outer membrane to promote glycolysis [60–62]. Pyruvate produced by glycolysis enters the mitochondria and is converted into acetyl-CoA as a substrate for the TCA Cycle. In addition, the interaction between Akt and hexokinase may inhibit the release of cytochrome c and maintain the structural and functional integrity of mitochondria, thus inhibiting neuronal apoptosis [63, 64]. In addition, Akt can also regulate mitochondrial biogenesis by regulating PGC-1α expression via mTOR to control mitochondrial oxidation [65, 66]. insulin signalling can also inhibit forkhead box O 1 (FOXO1) to inhibit the expression of heme oxygenase-1, which oxidizes heme to bilirubin and free Fe^3+^ to affect the activity of mitochondrial electron transport chain (mETC) and reduce NAD/NADH ratio and ATP production [59, 67]. Indeed, mitochondrial dysfunction, including reduced mitochondrial membrane potential, decreased mETC activity, reduced ATP production, and increased ROS, have been observed in T2DM and in insulin-resistant brains, while improved insulin signaling reversed these changes and improved synaptic plasticity and cognitive function [25, 68–70].

Neurodegenerative mechanism may work synergistically with T2DM to damage brain mitochondrial structure and function and cognition. During the development of brain insulin resistance, ceramide promote cleavage of amyloid beta precursor protein (APP) by β and γ-secretase to produce Aβ, which is the pathogenic molecule of AD [71, 72]. Meanwhile, high insulin levels in T2DM circulation can compete with Aβ for binding to insulin-degrading enzymes, reducing Aβ degradation [73]. Abnormal Aβ production and clearance will lead to its excessive accumulation in the brain. In turn, accumulated Aβ can compete with insulin for binding to the insulin receptor, reduce insulin receptor autophosphorylation, and decrease the affinity of insulin to its receptors, leading to disruption of insulin signaling [74]. Abeta oligomer also activates JNK leading to IRS-1 phosphorylation and degradation [75]. Therefore, a vicious circle may exist between insulin resistance and AD pathology. Elevated Aβ accumulates in synaptic mitochondria prior to extracellular accumulation, inhibiting mitochondrial respiration and biogenesis, resulting in overproduction of ROS, impaired mETC function, and altered calcium homeostasis [76–79].The increase of ROS in turn increases APP processing and Aβ production [80, 81].

Mitochondria are cellular energy factories. Cognitive function depends on the activity of neurons and synaptic connections, including the generation of action potentials, vesicle circulation, and neurotransmitter release [82, 83]. The high energy requirements generated by these processes and limited glycolysis capacity cause neurons to be extremely dependent on mitochondria [84]. Both insulin resistance and accumulation of Aβ lead to mitochondrial dysfunction, affecting energy supply to brain neurons, resulting in failure of neuronal metabolic control and promoting neurodegeneration [85–87]. In addition, the brain is highly vulnerable to oxidative stress due to its high rate of oxygen consumption and high levels of polyunsaturated fatty acids, coupled with low activity of antioxidant enzymes and high levels of pro-oxidative metal ions (such as Fe^2+^) [88, 89]. Mitochondria are also a major source and target of ROS, the initial form of ROS being superoxide (O_2_^·−^), which is later converted to hydrogen peroxide (H_2_O_2_). Mitochondrial dysfunction produces excessive ROS, reduces mETC activity and ATP synthesis [90]. Meanwhile, mitochondrial DNA (mtDNA) encoding respiratory chain complexes is susceptible to ROS, resulting in oxidative damage and mutation of mtDNA, which further damages the function of mETC and aggravates energy failure and oxidative stress [91]. In T2DM, enhanced mitochondrial ROS levels have also been observed to activate the apoptotic cascade by triggering the release of cytochrome c, leading to neuronal apoptosis and impaired cognition [92, 93]. In T2DM, oxidative stress also induces a novel form of iron-mediated cell death via phospholipid peroxidation, ferroptosis. In hippocampal neurons of db/db mice, upregulation of transferrin receptor 1 levels and decreased levels of ferroportin-1 and Ferritin heavy chain, decreased expression of mitochondrial ferritin and increased expression of mitoferrin were observed, suggesting hippocampal neuronal and mitochondrial iron overload [94, 95]. Excess Fe^2+^ can react with H_2_O_2_ generated to generate hydroxyl radicals (•OH) with stronger oxidative ability through Fenton reaction, and undergo lipid peroxidation reaction with unsaturated fatty acids [96]. Elevated mitochondrial ROS and decreased glutathione peroxidases activity lead to accumulation of lipid peroxides, which trigger ferroptosis and cognitive deficits in hippocampal neurons in T2DM and AD [94, 97].

Given the complex links between mitochondrial dysfunction and insulin resistance, impaired energy metabolism, accumulation of Aβ, and oxidative stress, mitochondrial dysfunction may be a bridge between T2DM and AD, leading to cognitive impairment.

### Mitochondrial Protein Quality Control

**Fig. 1:**
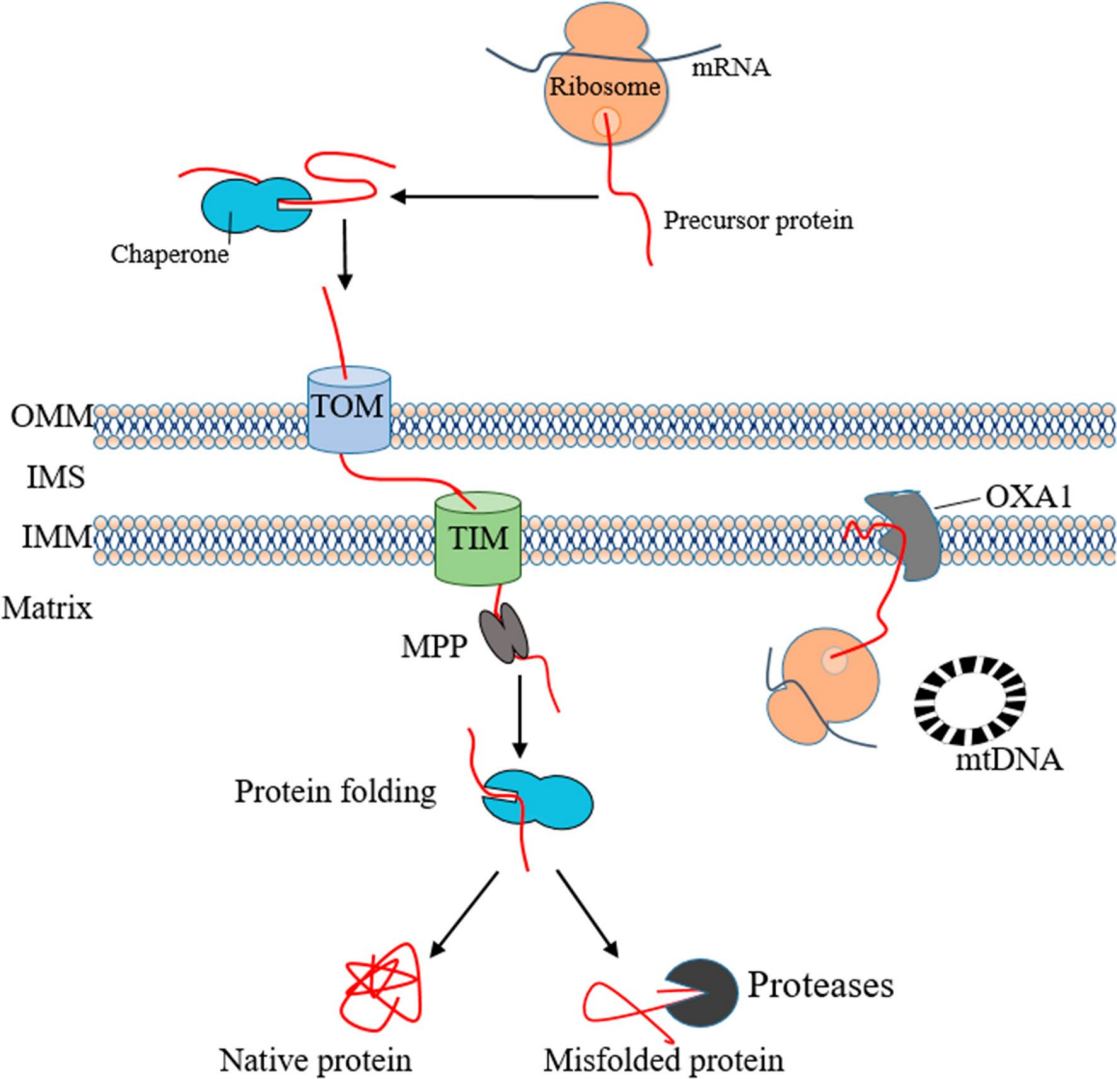
Mitochondrial protein quality control. Mitochondrial proteins encoded by nuclear genes are produced in the cytoplasmic ribosome, and molecular chaperones keep the precursor proteins unfolded. Precursor proteins pass through and enter the mitochondria through the TOM and TIM complexs on the OMM. After the precursor protein that enters the matrix is processed by MPP, the mitochondrial chaperones folds it to maturity. Misfolded proteins can be hydrolyzed by proteases. Mitochondrial-encoded proteins can be co-translationally inserted into the IMM via OXA1. *TOM* translocases of the outer mitochondrial membrane, *TIM* translocase of the inner membrane, *OMM* outer mitochondrial membrane, *IMS* intermembrane space, *IMM* inner mitochondrial membrane, *MPP* mitochondrial processing peptidase, *OXA1* oxidase assembly protein 1

In humans, only 13 proteins involving the subunit of respiratory chain complexes are encoded by the mitochondrial genome, while the remaining 1500 proteins are encoded by nuclear DNA [98]. Mitochondrial-encoded proteins can be inserted co-translationally into the inner membrane via the oxidase assembly protein complex [99, 100]. Precursor proteins encoded by nuclear DNA are produced on cytoplasmic ribosomes and subsequently imported into mitochondria in unfolded form with the help of molecular chaperones [101, 102]. With the support of mitochondrial membrane potential, precursor proteins are transported to the mitochondrial matrix through the translocase of the outer membrane complex on the outer mitochondrial membrane (OMM) and translocase of the inner membrane complex on the inner mitochondrial membrane (IMM) [103–106]. Precursor proteins entering the matrix are processed by mitochondrial processing peptidase, and then the molecular chaperones assists in folding imported proteins [107–110]. Misfolded or redundant proteins are degraded by ATP-dependent proteases [111–114]. When the accumulation of unfolded or misfolded proteins exceeds the cleaning capacity of mitochondria, the mitochondrial unfolded protein response (UPR^mt^) is induced [115]. In UPR^mt^, signals released from mitochondria trigger transcription of nuclear genes that encode mitochondrial chaperones and proteases to prevent harmful proteins from accumulating in the mitochondria [116–118]. Figure 1 illustrates the process of mitochondrial protein quality control.

Limited evidence shows defects in the quality control of mitochondrial proteins in diabetes. The levels of mitochondrial protease (Lon Peptidase 1) and mitochondrial chaperones (heat shock protein 60 and 70) were significantly reduced in the brain of T2DM mice, suggesting a deficiency of UPR^mt^ [119, 120]. Metformin can induce UPR^mt^ and significantly improve the function of brain mitochondria in T2DM mice [120]. In addition, the increased expression of heat shock protein 70 in the brain can improve insulin sensitivity and glycemic control [121]. Decreased mitochondrial chaperones heat shock protein 60 and 10 in the hypothalamus of T2DM lead to mitochondrial dysfunction and trigger neuronal insulin resistance, suggesting that defects in mitochondrial protein quality control may play an important role in the development of insulin resistance [122, 123]. However, the link between mitochondrial protein quality control system and cognitive impairment in T2DM remains largely unknown.

### Mitochondrial Biogenesis

**Fig. 2:**
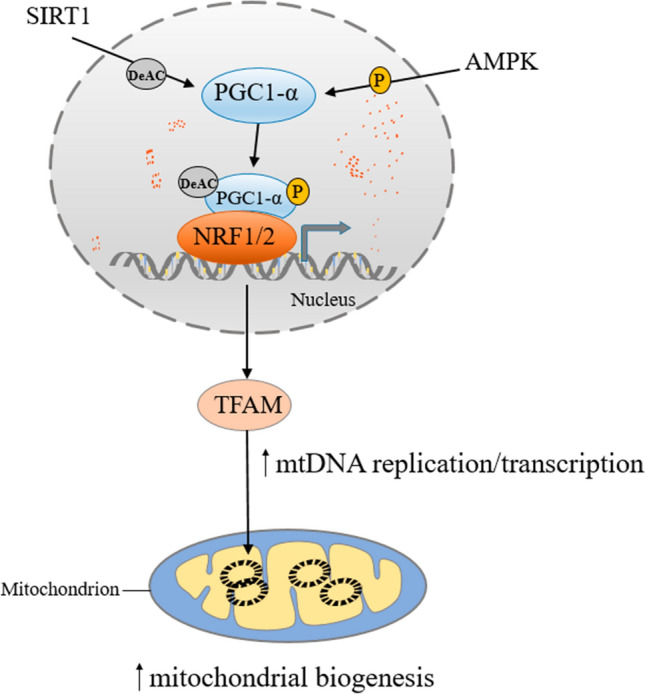
Mitochondrial biogenesis. Mitochondrial biogenesis is mainly regulated by PGC1-α. PGC1-α activates the expression of NRF1/2, thereby enhancing the expression of TFAM and promoting mitochondrial DNA replication and transcription. *PGC1-α* peroxisome proliferator-activated receptor-γ co-activator 1α, *NRF1* nuclear respiratory factor 1, *NRF2* nuclear factor erythroid 2-related factor 2, *TFAM* transcription factor A

Mitochondrial biogenesis is a compensatory response secondary to the damaged respiratory apparatus and low ATP production, aiming to replenish mitochondrial components. In neurons, mitochondrial biogenesis mainly occurs in soma [124]. Peroxisome proliferator-activated receptor gamma coactivator-1-alpha (PGC-1α) is regarded as the core of transcriptional control of mitochondrial biogenesis, activated by sirtuin 1 (SIRT1) and AMP-activated protein kinase (AMPK) induced deacetylation and phosphorylation, respectively [125]. It can augment the expression and activity of several critical transcription factors, including nuclear respiratory factor 1 (NRF1) and nuclear factor erythroid 2-related factor 2 (NRF2), peroxisome proliferator-activated receptor-α, oestrogen-related receptor-α and transcriptional repressor protein YY1 [126, 127]. Recent studies have found that NRF2 transcriptionally not only increases mitochondrial biogenesis, but also regulates mitochondrial genes in cooperation with PGC-1α. NRF2 can bind to PGC-1α to enhance gene induction of NRF1 and mitochondrial transcription factor A (TFAM), which is a key enhancer protein ensuring mtDNA replication by mtDNA polymerase γ [128–130]. These transcription factors can improve mitochondrial function and against oxidative stress and inflammation by regulating the expression of related proteins [131, 132]. Figure 2 illustrates the process of mitochondrial biogenesis.

The study showed that the expression of SIRT1 and PGC-1α in the brain of T2DM mice decreased significantly [120]. Decreased PGC-1α expression and activation in hippocampal neurons of T2DM mice leads to blocked mitochondrial biogenesis and mitochondrial dysfunction, triggers neuronal loss, and promotes cognitive impairment in diabetes [133, 134]. Downregulation of PGC-1α in the hippocampus of T2DM may result from dipeptidyl peptidase-4 binding to protease-activated receptor 2 and triggering glycogen synthase kinase-3β (GSK3β) activation [133]. Insulin signaling has also been found to be involved in regulating PGC-1α expression, affecting mitochondrial biogenesis [135, 136]. It was found that hyperinsulinemia caused by T2DM activates the hyperactivation of the insulin signaling factor Akt in the anterior cortex and hippocampus, resulting in the phosphorylation inactivation of FOXOs and subsequent reduction of PGC-1α, and accumulation of Aβ [137]. Interestingly, another study in UCD-T2DM rats showed that with the progression of brain insulin resistance, AMPK phosphorylation and SIRT levels in hippocampal neurons decreased, and mitochondrial biogenesis-related PGC-1α and TFAM expressions were significantly decreased, leading to increased lipid peroxidation and decreased synaptic plasticity in hippocampal neurons [138]. A study using primary rat cortical neurons also showed that palmitate induces neuronal insulin resistance and suppresses PGC-1α expression, contributing to mitochondrial dysfunction and decreased cell viability [139]. These studies suggest that PGC-1α has beneficial effects on diabetic brain neurons, and impaired insulin signaling may induce and exacerbate neuronal damage through inhibition of PGC-1α.

### Mitochondrial Dynamics

Mitochondria are highly dynamic organelles that continually undergo fusion and fission [140, 141]. Mitochondrial fusion promotes mixing of membranes and contents between mitochondria to supplement oxidative damage components, safeguard mtDNA integrity and preserve mtDNA function in the face of mutations [142, 143]. Mitochondrial fission contributes to the even partitioning of mitochondria to daughter cells during mitosis, and separation of damaged mitochondria for autophagic degradation [144, 145]. Two classes of dynamin-like protein are involved in mitochondrial fusion, including mitofusin (MFN) and optic atrophy 1 (OPA1). MFN mediate IMM fusion. MFN1 and MFN2 on the OMM of two adjacent mitochondria form both homooligomeric and heterooligomeric complexes to promote mitochondrial fusion, which depends on GTP hydrolysis, and can be mediated by GTPase domain [146, 147]. The fusion of IMM is mediated by OPA1. OPA1 is encoded by nuclear genes and introduced into mitochondria through the mitochondria protein quality control system [148]. The OPA1 entering the mitochondria is hydrolyzed by protease in the matrix to form long isoform and short isoform OPA1 [149]. the long isoform OPA1 is anchored on the IMM, and the short isoform OPA1 regulates the fusion activity by forming a complex with the long isoform OPA1 [146, 150]. In addition, OPA1 is also involved in the formation of mitochondrial cristae [151].

**Fig. 3:**
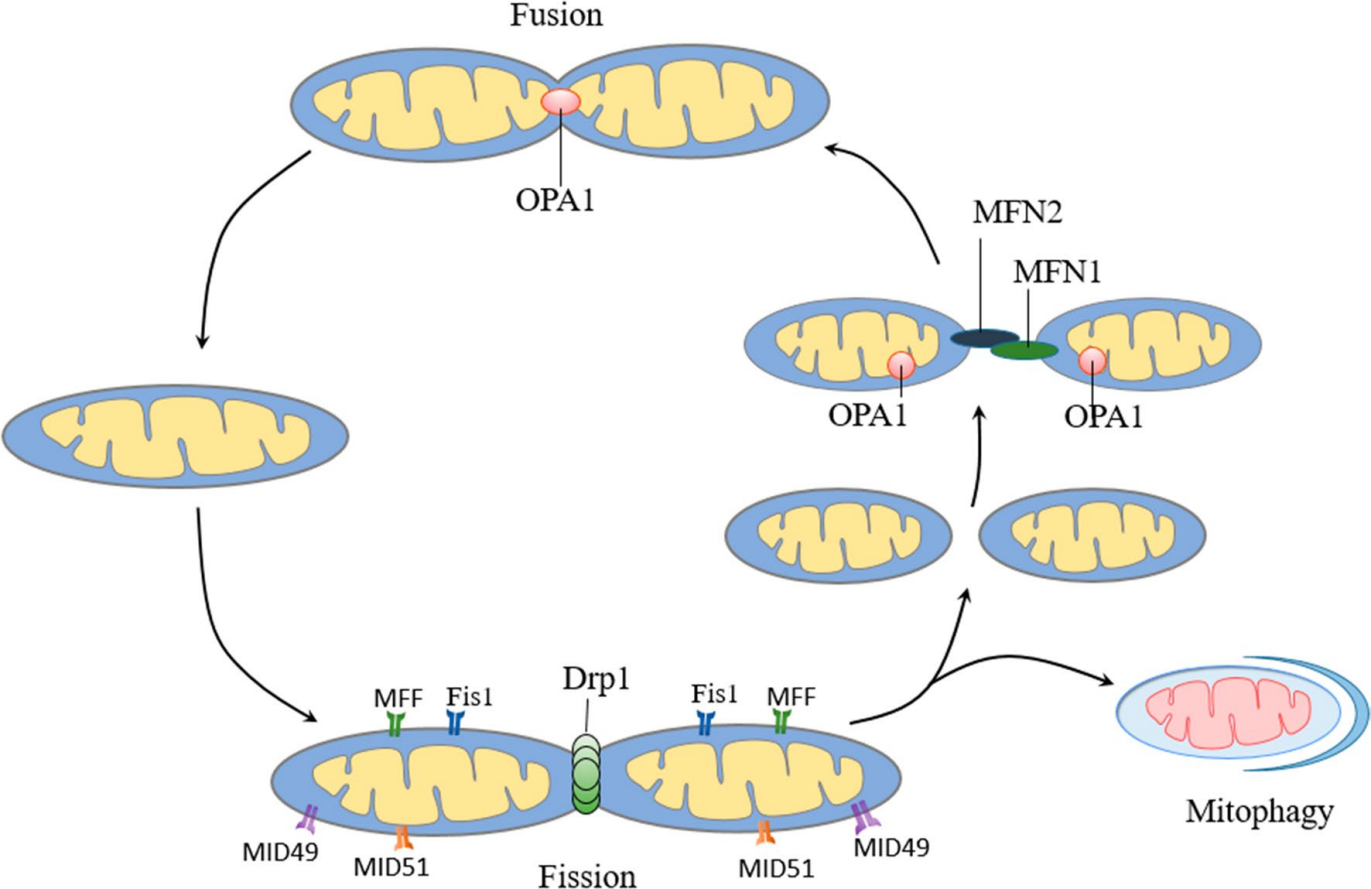
Mitochondrial fusion and fission. MFN1/2 interaction on adjacent mitochondria regulates OMM fusion. OPA1 mediates the fusion of IMM. Drp1 is recruited from the cytoplasm to the cleavage site of the OMM through its receptor, where it forms a ring-like structure to cleave mitochondria. *MFN1/2* mitofusin ½, *Drp1* dynamin-1-like protein

The key to mitochondrial fission is dynamin-related protein 1 (Drp1). Most mitochondrial fission occurs where ER tubules crossing or wrapping around. During fission, endoplasmic reticulum (ER) tubules mark sites of mitochondrial division and mediated constriction [152]. Then, Drp1 is recruited from the cytosol to the OMM at ER tubules mark sites by its receptors fission protein 1 (Fis1), mitochondrial fission factor (MFF), mitochondrial elongation factor 2 and mitochondrial elongation factor 1 [153]. Researchs suggest that although they all have the ability to recruit Drp1 to mitochondria alone, MFF and MID seem to be more important that their interplay could regulate Drp1 to promote mitochondrial fission [154, 155]. The recruited Drp1 molecules assemble into a ring-like structure to constricts and cleaves mitochondria by GTP hydrolysis [156]. Dynamin 2 also found to be involved in mitochondrial fission, but not necessary [157]. Figure 3 illustrates the process of mitochondrial fusion and fission.

In the hippocampus of type 2 diabetic mice and high glucose cultured human SK cells, GSK3β was activated to promote the significant expression and mitochondrial translocation of Drp1, which exacerbated mitochondrial fission and subsequently damaged the morphology and function of mitochondria [30]. Interestingly, levels of Mfn1, Mfn2, OPA1 were not altered in diabetic hippocampus, which was consistent with some research results [30, 69, 158]. However, some studies report that Opa1 was reduced in the cortex of GK mice with T2DM [159]. Knockout or inhibition of Drp1 significantly improves mitochondrial mass and reduces diabetes-induced hippocampal synaptic damage [30, 133]. However, it has been found that hippocampal neurons in neuron-specific Drp1-deficient T2DM mice exhibited marked mitochondrial dysfunction and synaptic damage, and higher levels of oxidative stress and neuroinflammation, which may be due to the fact that Drp1 knockdown inhibits mitochondrial fission and impairs the autophagy process [160]. In the hippocampal neurons of T2DM mice and PC12 cells cultured with high glucose, it was further found that promoting FUNDC1-mediated mitophagy can eliminate mitochondrial fragmentation caused by overactivated Drp1, reduce mitochondria-derived apoptosis, and thus alleviate diabetic cognitive impairment [161]. In addition, in vitro studies have shown that high glucose increases nitric oxide in cortical and hippocampal neurons in an N-methyl-D-aspartate receptor-dependent manner, leading to S-nitrosylation of Drp1, which leads to excessive mitochondrial division, impairs neuronal energy generation, and leads to synaptic loss and reduced plasticity [162]. These studies suggest that mitochondrial fragmentation owing to a loss of mitochondrial dynamics has a key role in the progression of cognitive impairment in diabetes.

### Mitophagy

**Fig. 4:**
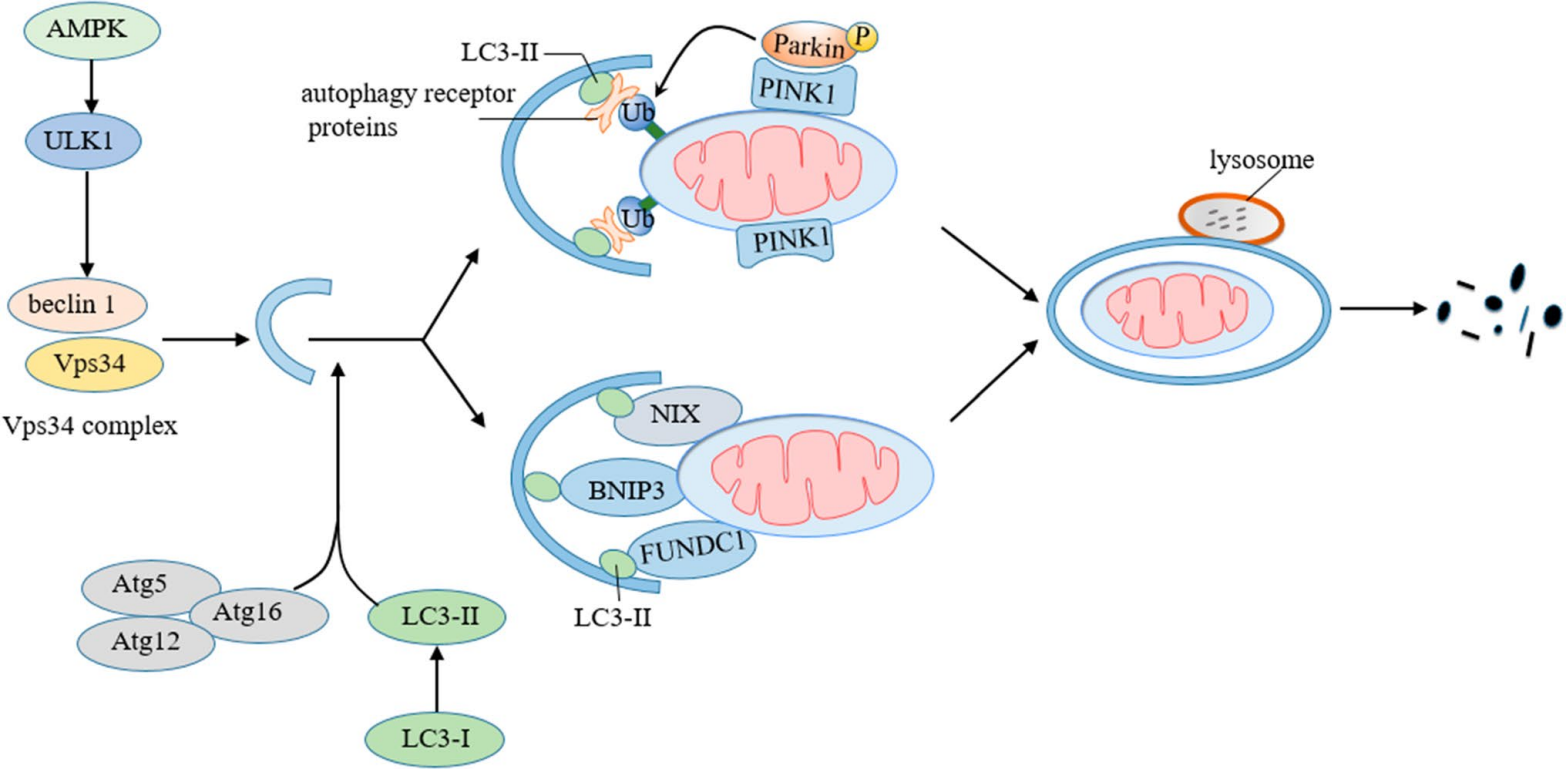
Mitophagy. ULK1 is activated by AMPK, induces nucleation of the phagophore by phosphorylating beclin 1 and activating Vps34 complex. Atg12-Atg5-Atg16 and LC3 further mediate autophagosome formation. LC3-I is processed into LC3-II and incorporated into the autophagosome membrane. Decreased mitochondrial membrane potential inhibits PINK1 entry into mitochondria, leading to PINK1 accumulation on OMM. Then, PINK1 recruits parkin from the cytoplasm to catalyze the formation of polyubiquitin chains on OMM proteins, which are then recognized by autophagy receptor proteins to form mitophagosome. Among other pathways of mitophagy, BNIP3, NIX and FUNDC1 in the OMM can directly bridge mitochondria to autophagosomes by interacting with LC3-II. *ULK1* Unc-51 Like Autophagy Activating Kinase 1, *AMPK* AMP-activated protein kinase, *Vps34* class III phosphatidylinositol 3-kinase, *LC3* light chain 3, *PINK1* PTEN- induced kinase 1, *BNIP3* BCL2/Adenovirus E1B 19 kDa Interacting Protein 3, *NIX* Nip3-like protein X, *FUNDC1* FUN14 domain containing 1

Mitophagy is a selective form of autophagy, that mediates the removal of defective and superfluous mitochondria. Mitophagy can promote mitochondrial turnover, avoid the accumulation of damaged mitochondria that can lead to cell degeneration, and adjust mitochondrial numbers to meet the energy demand [163]. Defects in mitophagy have been implicated in a variety of neurodegenerative diseases [164]. The process of mitophagy includes the detection and separation of damaged mitochondria, the recruitment of phagosomes and subsequent autophagic degradation [165]. Figure 4 illustrates the process of mitophagy.

Like other forms of autophagy, mitophagy is activated by phosphorylation of the Unc-51 like autophagy activating kinase 1 (ULK1) complex, typically by AMPK [166]. ULK1 induces nucleation of the phagophore by phosphorylating beclin 1 and activating class III PI3K complex [167]. The phagophore membrane extends and completely engulfs the target mitochondria and matures into an autophagosome mediated by Atg12-Atg5-Atg16 and LC3/Atg8 systems. During this period, the microtubule-associated protein 1 light chain 3 (LC3-I) is processed to LC3-II that is incorporated into the double membranes of the autophagosomes [168]. Then, the autophagosomes fuses with the lysosomes, resulting in the degradation of its cargo by acid hydrolases [169]. Several pathways have been found to detect target mitochondria and recruit autophagosomes for degradation to achieve the selectivity and specificity of mitophagy.

PINK1/Parkin-mediated mitophagy is the most well-known pathway. PTEN- induced kinase 1 (PINK1) is regulated by mitochondrial protein quality control mechanisms [99]. Under normal circumstances, PINK1 is introduced into IMM through TOM and TIM complexes, where it is cleaved by IMM protease presenilin-associated rhomboid-like, and then it is released into cytoplasm and degraded by proteasome [170]. The decrease of the membrane potential during mitochondrial dysfunction inhibits the entry of PINK1 into IMM through TIM complexes, resulting in the accumulation of PINK1 on the OMM.

On the mitochondrial surface, PINK1 recruits and phosphorylates Parkin to relieve its auto inhibited state and increase its E3 ligase activity [171]. PINK1 also phosphorylates Ubiquitin. Activated Parkin ubiquitylates multiple OMM proteins builds poly-ubiquitin chains on the OMM proteins, which in turn recruit autophagy receptor proteins, including p62, Next to BRCA1 gene 1 (NBR1), Nuclear domain 10 protein 52, Optineurin and Tax 1 binding protein 1 [172]. Autophagy receptor proteins simultaneously bind poly-ubiquitin chains in mitochondria through their ubiquitin binding domains, and LC3 on autophagosome membranes, which promotes the target mitochondria to be engulfed by autophagosomes, then autophagosomes fuse with lysosomes and lysosomal hydrolases degrade polyubiquitinated mitochondria [172, 173].

In addition to the classic PINK1/Parkin-related mitophagy, other autophagy receptors have also been found to regulate mitophagy, including Nip3-like protein X (NIX), BCL2/Adenovirus E1B 19 kDa Interacting Protein 3 (BNIP3), FUN14 domain containing 1 (FUNDC1). Under hypoxia, the expression of BNIP3 can significantly up regulate through hypoxia-inducing factor-1α, the inactive monomer BNIP3 in the cytosol forms a stable homodimer and is anchored to the OMM via its C-terminal transmembrane domain [174, 175]. The homodimer of BNIP3 can interact with LC3, which is further regulated by Ser17 and Ser24 phosphorylation near the LIR motif [176]. BNIP3 and NIX are proteins with homology to BCL2 in the BH3 domain. Similar to BNIP3, NIX is integrated into the OMM through dimerization and then binds to LC3 [177]. Several studies have reported that BNIP3 and NIX are also involved in PINK1/Parkin mediated mitophagy. NIX is ubiquitylated by Parkin, which promotes the recruitment of NBR1 to mitochondria [178]. BNIP3 can inhibit PINK1 proteolysis and promote its accumulation on OMM [179]. Of note, BNIP3 and NIX can also induce cell death [180]. FUNDC1 is an OMM protein that mediates hypoxia-induced Parkin-independent mitophagy by directly binging to LC3 [175, 181]. Under basal conditions, the activity of DC1 binding to LC3 is inhibited by phosphorylation of casein kinase 2 at serine 13 and SRC kinase at tyrosine 18 [182]. When encountering hypoxia, FUNDC1 is dephosphorylated by phosphoglycerate mutase family member 5 (PGAM5) at serine 13 and phosphorylated by ULK1 at serine 17, which increases interaction with LC3 to promote mitophagy [183, 184]. In addition, FUNDC1 interacts with OPA1 under normal conditions, while under mitochondrial stress, this interaction is reduced and promotes Drp1 recruitment to mitochondria [185]. It further reveals the “coupling” mechanism between mitochondrial dynamics and mitophagy.

In vitro models of diabetes, mitophagy of SK-N-MC and SH-SY5Y cells is significantly triggered in response to mitochondrial dysfunction and apoptosis induced by high glucose. The mitophagy seems to depend on PINK1 rather than BNIP3 or NIX, and has the effect of protecting neuronal cells. Melatonin could enhance PINK1-dependent mitophagy via the MT2/Akt/NF-κB pathway, thereby preventing ROS accumulation and antiapoptosis in neuronal cells under high glucose conditions [186]. However, some research found that LC3-II and p62 increased and PINK1 decreased in the midbrain of diabetic mice, indicating that autophagic flux is blocked. This was also seen in neuron-like PC12 cells cultured in high glucose. High glucose significantly blocked autophagic flux and inhibited PINK1/Parkin mediated mitophagy to reduced the viability of PC12 cells [187]. Significantly, these studies suggest that the enhancement of PINK1/Parkin mediated mitophagy is an important way to rescue neuronal cells in diabetes [186, 187]. Recent studies have also found the role of FUNDC1 related mitophagy in diabetic cognitive impairments. In T2DM mice, the dephosphorylation of FUNDC1 was inhibited, which promoted oxidative stress and neuroinflammation, resulting in apoptosis of hippocampal neurons. Similarly, the destruction of autophagic flux and the inhibition of FUNDC1-dependent mitophagy induced by high glucose exacerbated the apoptosis of PC12 cells. Activation of the Rac1/ROS axis appears to be an effective approach to prevent hyperglycemia-induced neurotoxicity by modulating FUNDC1-dependent mitophagy [161].

## Conclusion and Future Directions

Mitochondrial quality control is critical for the homeostasis of the mitochondrial network. The damage to multiple control mechanisms, such as imbalanced mitochondrial dynamics, impaired mitophagy and proteostasis disorder, was observed in diabetic cognitive impairment. However, the relative contribution of each dysregulated mechanism to cognitive impairment in diabetes is still unclear. In addition, because the mitochondrial quality control mechanism is a complex integrated hierarchical network of pathways, the changes of different quality control mechanisms can affect each other, and then alter the results of quality control. For example, proteins produced by mitochondrial biogenesis require the mitochondrial protein quality control system for import into mitochondria and proper assembly, and inhibition of mitochondrial fission might damage mitophagy and mitochondria biogenesis. Although current studies have noted that diabetes could affect interactions between mitochondrial quality control mechanisms, the functional consequences of these interactions are not fully understood and requires further experiments to determine the exact nature of their interplay. In conclusion, dissecting the mitochondrial quality control mechanisms and their interaction might be exploited to unveil new pathways for the prevention and treatment of diabetic cognitive impairment.

## Data Availability

The datasets generated during or analysed during the current study are available from the corresponding author on reasonable request.
